# A Review on Farnesoid X Receptor (FXR) Modulators Focusing on Benzimidazole Scaffold

**DOI:** 10.3390/molecules31030450

**Published:** 2026-01-27

**Authors:** Naoki Teno, Keigo Gohda, Ko Fujimori

**Affiliations:** 1 Faculty of Clinical Nutrition, Hiroshima International University, Kure 737-0112, Japan; 2 Computer-Aided Molecular Modeling Research Center Kansai (CAMM-Kansai), Nishinomiya 663-8241, Japan; ke.gohda@camm-kansai.org; 3 Department of Pathobiochemistry, Faculty of Pharmacy, Osaka Medical and Pharmaceutical University, Takatsuki 569-1094, Japan; ko.fujimori@ompu.ac.jp

**Keywords:** farnesoid X receptor (FXR), benzimidazole, FXR agonists, FXR partial agonists, dual modulators, FXR antagonists, intestinal FXR

## Abstract

The discovery of a mechanism by which bile acids (BAs) regulate fat synthesis by modulating the activation of the farnesoid X receptor (FXR) in the liver and intestines has highlighted the central role of BAs in triglyceride synthesis in the liver. FXR has been reported as a promising drug target for primary biliary cholangitis, metabolic-dysfunction-associated steatohepatitis, and metabolic-dysfunction-associated steatotic liver disease. A large number of FXR modulators with various chemotypes have been developed by many research groups. Although several FXR modulators are advancing into clinical trials, ongoing efforts aim to develop new FXR modulators that minimize the adverse effects associated with long-term administration. To develop drug candidates targeting FXR, various heterocyclic and/or fused heteroaromatic rings have been employed as the core and/or parts of the structures, out of which benzimidazole has been recognized as a valuable structural motif due to its synthetic accessibility and its versatility in constructing structurally diverse target molecules. Herein, we report on the development of FXR modulators incorporating benzimidazole as a fused heteroaromatic ring.

## 1. Introduction

**Figure 1:**
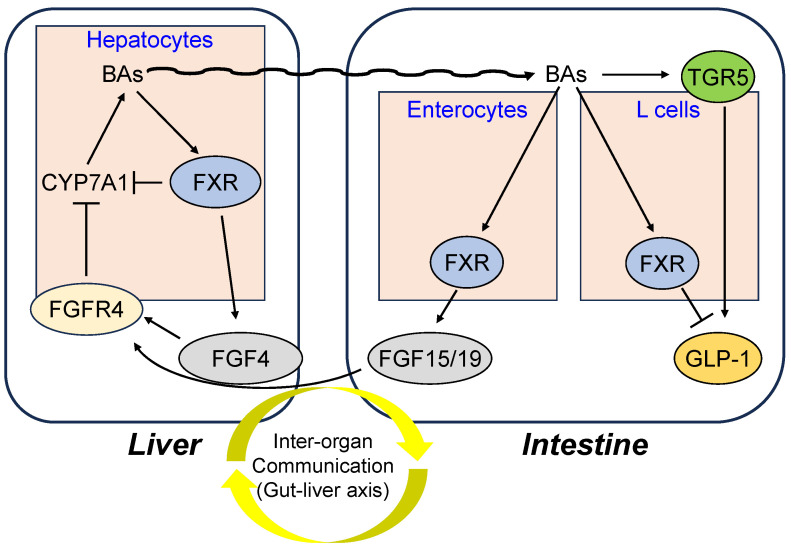
The role of FXR in the gut–liver axis. FXR contributes to crosstalk between the liver and gut by regulating BA synthesis in the liver and FGF15/19 and FGF4 secretion from the intestine. FGF15/19 and FGF4 regulate BA production through FGFR4 signaling in the liver. BAs function TGR5 as well as FXR and contribute to metabolic homeostasis. Arrows indicate the activating effects, whereas T bars indicate the inhibitory effects.

Farnesoid X receptor (FXR), a member of the nuclear receptor (NR) superfamily, has been identified as a bile acid (BA)-binding transcription factor and is a crucial regulator of bile acids, lipids, amino acids, and glucose homeostasis, as well as hepatic inflammation, regeneration, and fibrosis [1,2]. FXR is expressed at high levels in the digestive tract, which is responsible for enteric circulation [3]. Activation of FXR as a BA sensor by high levels of the primary BA, chenodeoxycholic acid (CDCA) [2], provides negative feedback on cholesterol and BA synthesis by inducing the expression of small heterodimer partner (SHP), leading to the inhibition of the expression of cholesterol 7α-hydroxylase (CYP7A1), the rate-limiting enzyme in BA synthesis. The expression of CYP7A1 is directly controlled by fibroblast growth factor 15/19 (FGF15/19) upon FXR stimulation, and FGF15/19 controls BA production via hepatic FGF receptor 4 (FGFR4) signaling [4]. Moreover, BA homeostasis in hepatocytes is controlled through the FXR-FGF4-FGFR4 axis [5]. FXR may play a pivotal role in the inter-organ network, as it is involved in the regulation of enterohepatic circulation of BAs. BAs activate both FXR and transmembrane G protein coupled receptor-5 (TGR5) to control BA homeostasis and glucose metabolism [6]. (Figure 1) BAs induced TGR5 gene expression to stimulate glucagon-like peptide-1 (GLP-1) production and improve hepatic glucose and lipid metabolism in high fat diet (HFD)-induced obese mice [7]. Intestinal BAs promote the secretion of FGF15/19 to regulate liver regeneration via hepatic FGFR4 [8,9]. Thus, because of the important role of FXR as an enterohepatic regulator, FXR ligands have attracted attention as a potential treatment for diseases triggered by metabolic abnormalities such as obesity, diabetes, hepatic disease, and chronic intestinal inflammation [10].

**Figure 2:**
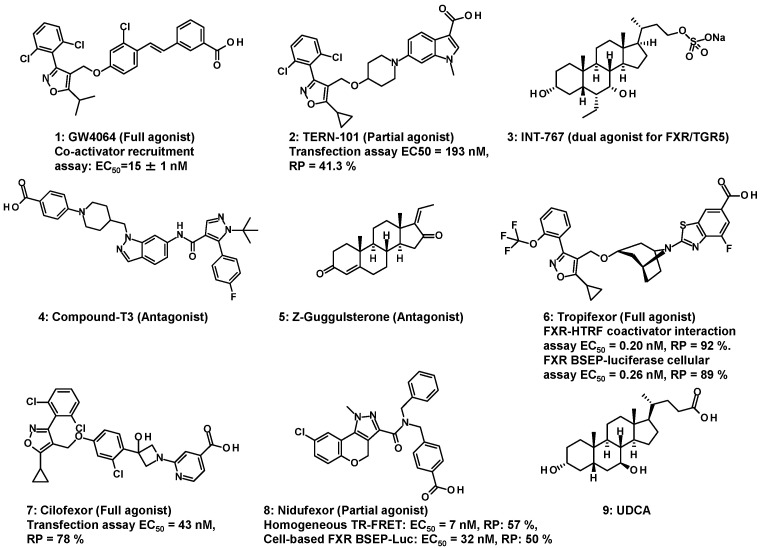
Various chemotypes of FXR modulators.

Accordingly, various FXR modulators with different functions—such as FXR agonists, partial agonists, dual modulators, and antagonists—have been reported. These modulators exhibit diverse chemotypes, as depicted in Figure 2. GlaxoSmithKline developed a non-steroidal full agonist GW4064 (**1**) with two structural characteristics: a substituted isoxazole moiety (commonly referred to as a hammerhead) and stilbene [11]. Terns Pharmaceuticals Annual Report 2023 identified TERN-101 (LY2562175) (**2**) as an FXR partial agonist that possesses indole [12]. Further, a hydrophilic steroidal dual agonist, INT-767 (**3**), for FXR/TGR5 was identified as a semi-synthetic BA derivative [13]. Takeda Pharmaceuticals developed a non-steroidal potent antagonist compound-T3 (**4**). This compound possessed indazole as the fused aromatic ring [14]. Similarly, in another study, steroidal guggulsterone (**5**), obtained from an herbal extract from the guggul tree, was identified as a naturally occurring antagonist of FXR [15].

A wide variety of FXR modulators have been reported in previous studies. Some of the FXR modulators are found to be associated with primary biliary cholangitis (PBC), metabolic-dysfunction-associated steatohepatitis (MASH), and metabolic-dysfunction-associated steatotic liver disease (MASLD) [12,16,17,18]. Clinical trials of tropifexor (LNJ452) (**6**) [17,18], cilofexor (**7**) [19,20,21], TERN-101 (LY2562175) (**2**) [22], and nidufexor (LMB763) (**8**) [16] as FXR full or partial agonists are underway to treat MASLD, MASH, or diabetic nephropathy. In recent years, combination studies of these agonists with other drug candidates have been investigated. For instance, LYS006 (inhibitor of leukotriene A4 hydrolase) [23] and tropifexor (**6**) therapy were well tolerated, with the exception of a high frequency of pruritus in the combination arm [24]. TERN-101 (**2**) is also undergoing a combination study with TERN-501 [25], a thyroid hormone receptor (THR) β agonist [26]. In contrast, a clinical trial of guggulsterone (**5**) failed to show any effect on low-density lipoprotein (LDL) cholesterol [27]. However, ursodeoxycholic acid (UDCA) (**9**), which has only low affinity for FXR and therefore lacks FXR agonistic activity [28], is commercially available to improve liver function in patients with chronic liver disease. Steroidal treatment (**9**) did not show any histological improvement after two years in a clinical trial for MASH [29].

Subsequently, the pharmacological and clinical knowledge of FXR modulators has been accumulating, and a wide range of pharmacophores, such as **1**–**9**, are being developed and reported. They are classified as either steroidal or non-steroidal. Non-steroidal modulators currently represent the main trend in the development of synthetic modulators, likely owing to the strategic focus of the research groups and the ability to rapidly adapt their structures to address emerging challenges. Even among non-steroidal modulators, some have the isoxazole moiety, such as GW4064 (**1**), while others have structures entirely different from that of GW4064 (**1**). The former includes compounds **1**, **2**, **6**, and **7**, whereas representative examples of the latter are **4** and **8**.

The building blocks of these structures include various heterocyclic and/or fused heteroaromatic rings. Indeed, many reviews have been published presenting a wide variety of FXR modulator structures, along with their associated biological and pharmacological profiles [30]. Of fused aromatic rings, benzimidazole is a fusion of a benzene and imidazole ring system at the 4- and 5-positions of the imidazole ring, and it has synthetic versatility and availability as starting materials. The characteristics of this ring system are (A) both acid and base properties, (B) the ability to form salts, and (C) conformation-restricted motif [31]. Thus, benzimidazole is a versatile core structure that is widely used as a basic motif in chemicals and as an alternative for various building blocks, making it a popular structural component of pharmaceuticals [31,32]. It is of particular interest to medicinal chemists and other communities working on FXR to understand how benzimidazole-containing compounds are being used in the development of modulators for FXR or other NRs. In this review, we have specifically focused on the application of benzimidazole for FXR.

## 2. Benzimidazole as Structural Motif in Medicinal Chemistry

**Figure 3:**
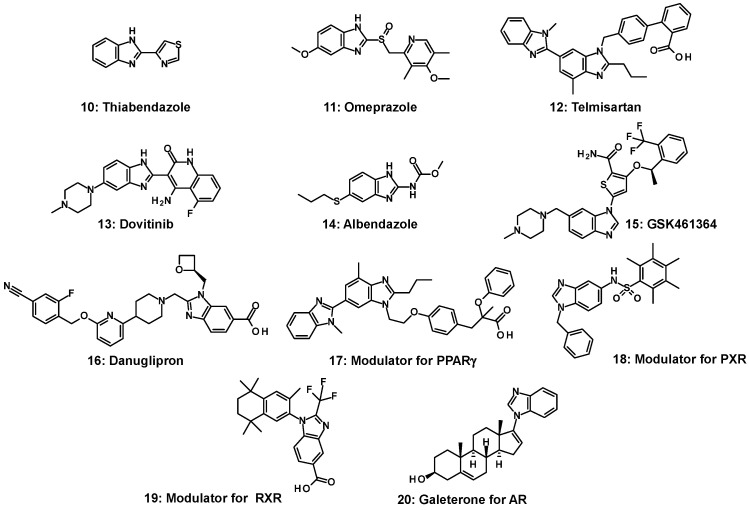
Benzimidazole as a structural motif in medicinal chemistry (**10**–**20**).

The structural motifs of fused heteroaromatic derivatives have been described as prominent structures; in particular, nitrogen-based heterocycles with a wide range of activities are used in the pharmaceutical, agricultural, and industrial fields [33]. In 1944, Woolley reported that benzimidazoles could cause the same biological reactions as adenine and guanine due to their structural similarity to purines. This discovery initiated diverse lines of research into benzimidazole derivatives [34]. Naturally occurring vitamin B_12_, abundant in seafood, algae, meat, eggs, and milk, contains a 5,6-dimethyl benzimidazole moiety [35]. Benzimidazole is an integral part of vitamin B_12_ and also serves as a core structure in benzimidazole nucleosides and nucleotides [36]. The chemistry of benzimidazoles was first described in 1951 [35]. As a result of these early studies, thiabendazole (**10**) was introduced to the market as an anthelmintic drug approximately 50 years ago [37]. (Figure 3) Since then, substituted benzimidazoles have played significant roles in drug discovery across various therapeutic areas: omeprazole (**11**) as a gastric ulcer agent [38], telmisartan (**12**) for the treatment of hypertension [39], dovitinib (**13**) as a suppressor of lung metastasis [40], and albendazole (**14**) as a broad-spectrum anthelmintic [41].

Benzimidazole-based compounds interact with enzymes and receptors through multiple binding modes. Most of these compounds are highly selective ATP-competitive inhibitors [42]. Benzimidazole and related heteroaromatic motifs of GSK461364 (**15**), an inhibitor of polo-like kinase 1, have been widely reported as kinase-targeting scaffolds that form hydrogen bonds with the hinge region of kinases [43]. These are currently in clinical trials for non-Hodgkin’s lymphoma treatment [44]. Most recently, Pfizer designed a non-peptide GLP-1 agonist, danuglipron (**16**), using a benzimidazole derivative, which is undergoing clinical trials as an oral treatment for type 2 diabetes mellitus (T2DM) [45].

Benzimidazole contributes to the development of modulators of the peroxisome proliferator-activated receptor (PPAR) γ (**17**) [46] and pregnane X receptor (PXR) (**18**) [47], which belong to the same NR1 subfamily as FXR. With respect to the retinoid X receptor (RXR) and androgen receptor (AR), which are classified into NR2 and NR3 subfamilies, respectively, the modulators of RXR (**19**) and AR (**20**) also have benzimidazole moieties [48,49]. In particular, steroidal galeterone (TOK-001) (**20**) [48] with benzimidazole, developed by Tokai Pharmaceuticals, is a selective CYP17A1 inhibitor and AR antagonist.

As a summary, from the mid-1900s to the present, benzimidazole substituents and substitution positions significantly affect the biological activity and physicochemical properties of benzimidazole derivatives and are therefore crucial in the development of pharmaceuticals.

## 3. FXR Agonists

### 3.1. FXR Full Agonists with an Isoxazole Moiety and No Benzimidazole

GW4064 (**1**) was developed based on the lead compound obtained from an in-house combinatorial library consisting of 9900 compounds [11]. (Figure 2) The isoxazole moiety of **1** has exerted a major impact not only on the structure of subsequent FXR agonists but also on the partial structure of antagonists. In contrast, the stilbene moiety of **1** is a potentially toxic pharmacophore owing to the conformational changes caused by UV radiation, making further development less likely [50,51]. One approach to exploring FXR agonists derived from **1** is to connect a trisubstituted isoxazole moiety and an arylcarboxylic acid moiety with a suitable linker, resulting in the entire molecule occupying the ligand-binding domain (LBD) of FXR [17,21,50,51]. The following representative FXR full agonists were obtained as a result of the linker exploration.

Tropifexor (**6**) [17] and cilofexor (**7**) [21] have the isoxazole moiety and unique structural motifs, including aryl carboxylic acid: bicyclic nortropine-substituted benzothiazole carboxylic acid and a hydroxyazetidinyl linker as a surrogate for the stilbene moiety, respectively.

### 3.2. FXR Partial Agonists

*Partial agonists with an isoxazole moiety and without benzimidazole*: TREN-101 (LY2562175) (**2**) is constructed with a substituted isoxazole moiety and piperidine and indole as replacements for the stilbene in **1** [22]. (Figure 2) The biological activity of **2** has a higher EC_50_ value than that of **1** [12]. Some FXR agonists developed to date have been reported to improve metabolic liver disease, but they are also associated with adverse effects in clinical testing, such as pruritus, lowering high-density lipoprotein (HDL) cholesterol, and increasing LDL cholesterol [52]. Partial activation could be a promising approach that targets FXR, with the hope that it may reduce the side effects, as suggested by encouraging clinical results on **2** [22]. Nevertheless, it remains unclear whether partial FXR activation can indeed limit some of these side effects.

**Figure 4:**
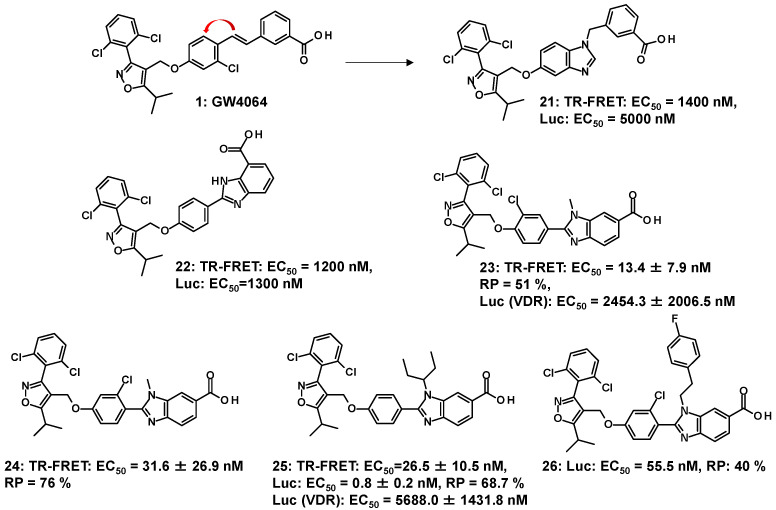
Progress from GW4064 (**1**) to **21** and agonists (**21**–**26**) with isoxazole moiety and benzimidazole.

**Figure 5:**
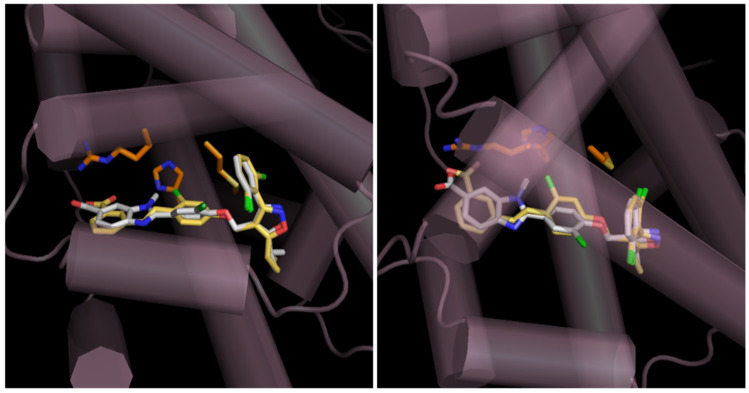
Modeling of FXR complexed with GW4064 (**1**) and **23**. A complex model of human FXRα-LBD monomer (PDB ID: 3DCT, purple cylinders and orange sticks) with GW4064 (**1**) (X-ray; yellow sticks) and **23** (model; white sticks) was built using AutoDock Vina1.1.2. Left panel: view from the side. Right panel: view from above. Reprinted with permission from Ref. [53].

*Partial agonists possessing an isoxazole moiety and benzimidazole*: The GlaxoSmithKline group has shown that the central aromatic ring of **1** is linked to the distal (red arrow) stilbene carbon to yield benzoimidazole-containing **21** with a rotatable carbon (Figure 4); however, the agonist activity of **21** was significantly lower than that of **1** [50]. Since the report on **21**, the synthesis of many FXR agonists with fused aromatic rings replacing the stilbene moiety has begun, as shown in the following **22**–**26**. The elimination of the stilbene moiety via conformational constraints while sustaining the trisubstituted isoxazole moiety resulted in the production of **22** with benzimidazole [51]. Compound **22** has been investigated for the synthesis of many derivatives. Such modulators (**23**–**26**) [53,54,55] are shown in Figure 4. Like **21**, N-substituted **23** and **24** are effective at double-digit nM in the TR-FRET assay; however, both analogs have 51% and 76% RP to **1**, showing that **23** and **24** partially activate FXR. Molecular modeling of **23** (white sticks in Figure 5) into the LBD of FXR supported the idea that the isoxazole moiety and the carboxylic-acid-bearing pharmacophore in **23** overlapped well with those of **1** (yellow sticks in Figure 5), and the benzimidazole moiety in **23** and the stilbene moiety in **1** shared the same binding site in FXR, resulting in **23** showing the same binding mode as GW4064 (**1**) [53]. (Figure 5) Compound **23** showed slight affinity to vitamin D receptor (VDR) in addition to FXR in NR1 [55].

Additionally, FXR activation by **23** increases the bone morphogenetic protein-2-induced differentiation of mesenchymal stem cells (MSCs) into osteoblasts through the activation of runt-related transcription factor 2 expressions. This suggests that FXR agonists may also be involved in the treatment of bone diseases such as osteoporosis [53]. The branched bulky substituent of **25** revealed a higher EC_50_ value than **23** and slightly reduced VDR activity while retaining its partial agonist properties [55]. The accumulated structure–activity relationship (SAR) data on the analogs with a benzimidazole scaffold were analyzed by molecular dynamics simulation, and **26** with N1-substituted benzimidazole as an FXR partial agonist was designed based on the results [54].

**Figure 6:**
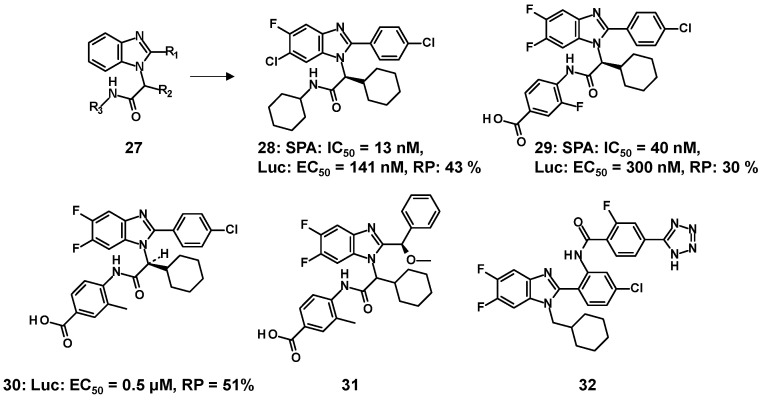
FXR partial agonists (**28**–**33**) with benzimidazole scaffold and no isoxazole moiety.

*Partial agonists with benzimidazole and no isoxazole moiety*: In 2011, Hoffmann-La Roche assembled an in-house library with a benzimidazole core and developed compounds that retained the benzimidazole even in the hit-to-lead process [56]. The pharmaceutical company discovered derivatives of benzimidazolyl acetamides, represented by the general formula (**27**), with FXR agonist activity through a scintillation proximity assay (SPA) using the LBD of human FXR. (Figure 6) Compound **28** was found from an in-house library centered on benzimidazole. Consequently, **28** was identified as a lead compound in the hit-to-lead process [56]. Following this, in vivo studies of **28** were conducted using LDL-receptor-knockout mice after examining its physicochemical properties and pharmacokinetics, showing that **28** reduced the level of total cholesterol, LDL, and triglycerides [57]. Lead optimization efforts proceeded without changing the core structure of the benzimidazole moiety. As a further modification of the benzimidazole scaffold, multiple halogen atoms were introduced onto benzimidazole; additionally, the R1 to R3 components were changed in **27** to overcome the drawbacks (e.g., solubility and bioavailability) of **28**. The structure-based drug design provided orally available **29** with favorable pharmacokinetic properties in rodents and plasma-lipid-declining effects in LDL-receptor-knockout mice [56]. Partial agonists (**30**–**32**) derived from **28** and **29** were covered by the patents [58,59].

**Figure 7:**
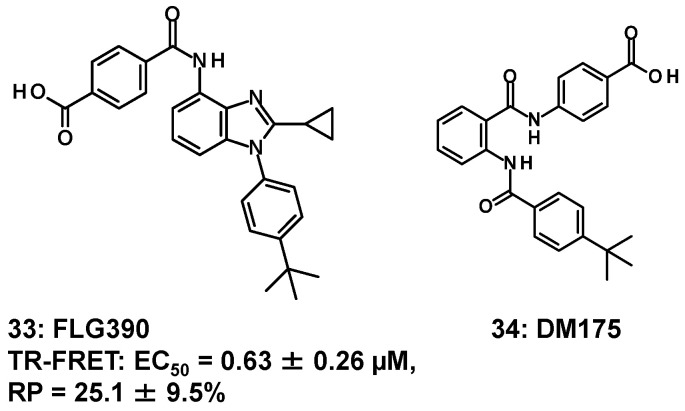
FXR partial agonists (**33**, **34**) other than **28**–**32**.

Unlike **28**–**32**, trisubstituted benzimidazole such as **33** can be obtained by selecting different starting materials [60]. (Figure 7) The binding ability of **33** was validated by a TR-FRET assay, and **33** revealed no affinity (less than 5%) to any members of the NR superfamily other than FXR. In addition, as mentioned in the study using **23**, it was found that **23** promotes differentiation of MSCs into osteoblasts. Furthermore, Fujimori et al. used **33** to elucidate the mechanism of FXR activation, where it was found that **33** activates prostaglandin E2 produced by cyclooxygenase-2 at the early stage of MSC differentiation into osteoblasts via the EP4 receptor [60].

Many of the benzimidazole-containing derivatives shown here, including trisubstituted benzimidazole **33**, can be synthesized essentially by the synthetic method depicted in Scheme S1 (Supplementary Materials).

*Partial agonists without an isoxazole moiety and benzimidazole:* The Genomics Institute of the Novartis Research Foundation identified the FXR partial agonist nidufexor (LMB763) (**8**) [16] to explore a novel chemical pharmacophore by high-throughput screening (HTS) using a library composed of approximately 3 million compounds. As depicted in Figure 2, the tricyclic dihydrochromenopyrazole core in nidufexor (**8**) is a very unique pharmacophore among FXR modulators.

The design and synthesis of partial agonists with an anthranilic amide moiety as the core structure began in 2014 [61], and in 2019, DM175 (**34**) (Figure 7) was reported to have in vivo activity [62]. The complex with the benzimidazole-bearing partial agonist (**30**) [57] seemed most suited for docking studies on the derivative of **34** [61].

Compounds **8** and **34** have no benzimidazole scaffold and are thereby considered to be out of the scope of this review. However, we include them since they are crucial FXR agonists in the FXR community; the former is under investigation for its clinical usefulness in trials [11,16], and the latter has shown in vivo activity [62] and has been employed for the mechanism analysis of partial agonists [63], as shown in the next subsection.

*Mode of action of partial agonists*: A conformational change in FXR corresponding to ligand interactions results in corepressor dissociation, coactivator association, and the regulation of target genes [62]. However, the structural changes in the LBD caused by FXR partial agonists remain unclear.

Merk et al. demonstrated a possible mechanism of action for the FXR partial agonist DM175 (**34**) (Figure 7), showing that binding of **34** shifts Trp454, located on helix 11–helix 12 [activation factor 2 (AF2)] of FXR, outward via site occupancy by the *t*-butyl group of **34**. However, the naturally occurring primary bile acid, chenodeoxycholic acid, has no effect on the shifting of Trp454. DM175 (**34**) demonstrated the most striking difference in the complex structure with partial agonists [62]. In addition, the distinct molecular effects of several different scaffolds on FXR activation were apparent and consistent with the characteristic structural changes within the LBD of FXR, leading to partial FXR agonists inducing FXR-regulated gene expression, which is significantly different from the effects of FXR agonists [63].

The mechanism of the FXR partial agonist has been elucidated not only by non-benzimidazole derivatives but also by the compounds containing benzimidazole. SAR analysis of the compound cluster, including benzimidazole derivatives (such as **26**), showed that fluctuations in helix 8 of the LBD of FXR may influence its agonistic activity. The findings of this study may aid in designing novel FXR partial agonists [54].

Furthermore, Asthana et al. suggested that three methionine residues, namely Met328, Met365, and Met450, are essential for attaining FXR partial agonism using MD simulations, residue-wise communication network analysis, and thermodynamic profiling [64].

These reports on the molecular mechanisms underlying the partial agonist activity may provide important clues for minimizing interference with metabolic cholesterol degradation while maximizing agonism against FXR. Determining the critical residues in FXR that distinguish the binding modes of agonists from that of partial agonists is essential for designing future partial agonists.

## 4. Dual Modulators Focusing on Benzimidazole Scaffold for FXR and Other Target Molecules

**Figure 8:**
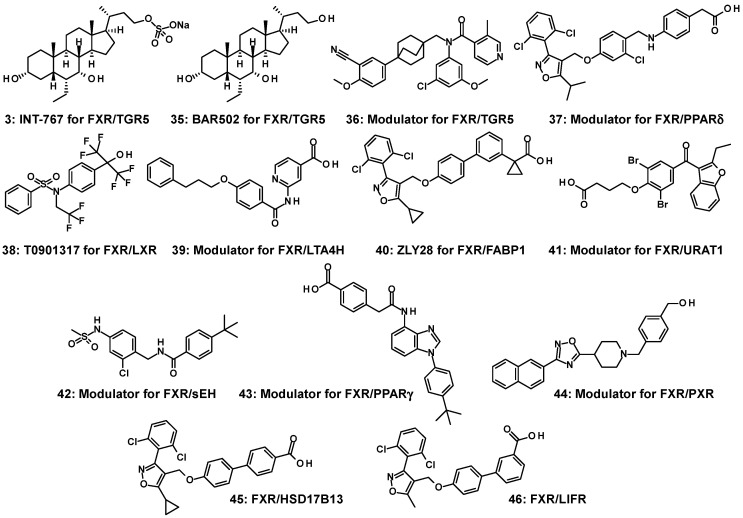
Reported dual modulators (**3**, **35**–**46**) for FXR and other target proteins.

Dual modulators for multiple target molecules have been developed considering their therapeutic rationality. From the perspective of pharmacotherapy, it seems reasonable to treat multi-factorial diseases such as MASH and MASLD using multiple and different pathological factors [65]. In this case, polypharmacy with a multitude of drugs may be considered as a treatment strategy; however, it also has disadvantages, such as complex and problematic drug–drug interactions and the adverse effects they may cause. Multi-targeted drugs that address possible therapeutic mechanisms have the potential to circumvent many of the drawbacks associated with polypharmacy [66]. The number of dual modulators for FXR and other target molecules is currently rising: [A] steroidal BAR502 (NorECDCOH) (**35**) [67], steroidal INT-767 (**3**) [13], and nonsteroidal **36** [68] for FXR/TGR5, [B] nonsteroidal **37** [69] for FXR/PPARδ, [C] nonsteroidal T0901317 (**38**) [70] for FXR/liver X receptor (LXR), [D] nonsteroidal **39** [71] for FXR/leukotriene A4 hydrolase (LTA4H), [E] ZLY28 (**40**) [72] for FXR/intestinal fatty acid binding protein 1 (FABP1), [F] nonsteroidal **41** [73] for FXR/urate transporter 1 (URAT1), [G] nonsteroidal **42** [74] for FXR/soluble epoxide hydrolase (sEH), [H] benzimidazole-containing **43** [75] for FXR/PPARγ, [I] nonsteroidal **44** [76] for FXR/PXR, [J] nonsteroidal **45** [77] for FXR/17-β-hydroxysteroid dehydrogenase 13 (HSD17B13), and [K] nonsteroidal **46** [78] for FXR/leukemia inhibitory factor receptor (LIFR). (Figure 8) Benzimidazole scaffolds are employed in the development of FXR/sEH and FXR/PPARγ dual modulators; however, there are differences in how they are utilized in each case.

**Figure 9:**
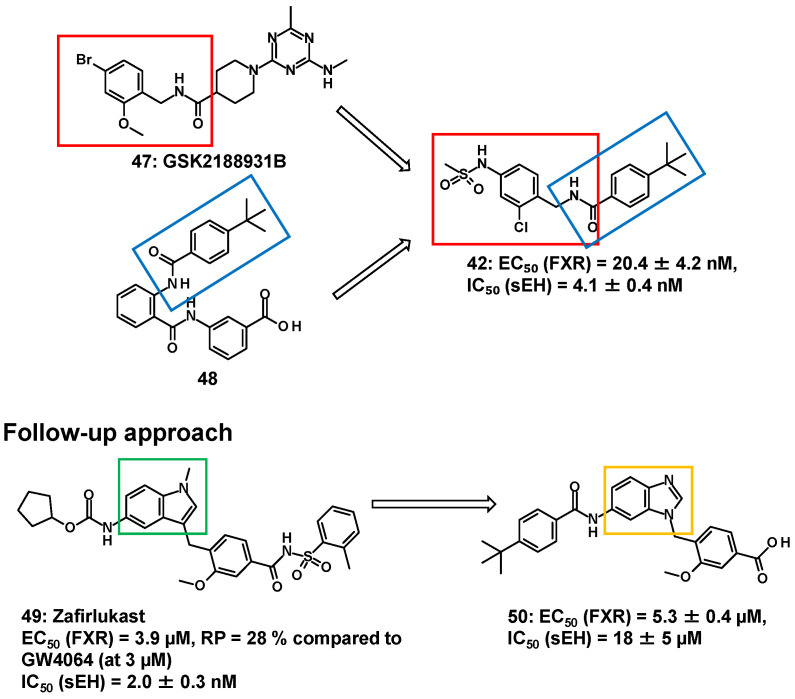
FXR/sEH dual modulator (**42**) and subsequent follow-up approach.

Merk et al. reported innovative multi-targeted agents that are dual modulators of FXR and sEH [74,79]. sEH, a downstream enzyme of CYP epoxygenase (CYP2C and CYP2J) of arachidonic acid metabolism, has shown promising results in the treatment of T2DM [80]. Since MASH is associated with many risk factors (e.g., T2DM), it seems reasonable to treat such multi-factorial diseases, which have distinct pathological factors exhibiting more than one therapeutic mechanism [65]. Based on the pharmacophore of GSK2188931B (**47**)—which includes an N-benzylamide residue (red square in Figure 9) for sEH inhibition [81]—and the *t*-butyl phenyl (blue square in Figure 9) of **48** for FXR activation [61], **42**, composed of N-benzylamide residue and *t*-butyl phenyl, demonstrated robust therapeutic efficacy in animal MASH models, along with improved aqueous solubility (1.5 μg/mL) [74]. Following this, zafirlukast (**49**), an anti-asthmatic drug with an indole, has a weak agonist activity against FXR (EC_50_ value of 3.9 µM and 28% RP) compared to GW4064 (at 3 µM), and it has a limited inhibitory effect against sEH. To combine the favored structural variations in the indole (green square in Figure 9) of **49**, the elements necessary for the sEH and FXR modulators were arranged on benzimidazole (yellow square in Figure 9); however, **50** was inferior to **49** in terms of the inhibitory activity against sEH [82]. For unknown reasons, no structural transformations of the scaffold other than benzimidazole have been investigated in the indole of **49**. Although a decrease in activity of **50** was observed, considering the SAR of the fused heteroaromatic rings, benzimidazole cannot be discounted.

**Figure 10:**
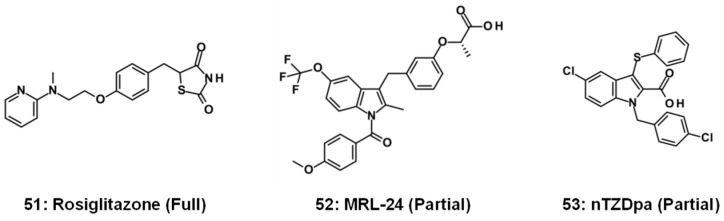
Previously reported PPARγ full agonist (**51**) and partial agonists (**52**, **53**).

**Figure 11:**
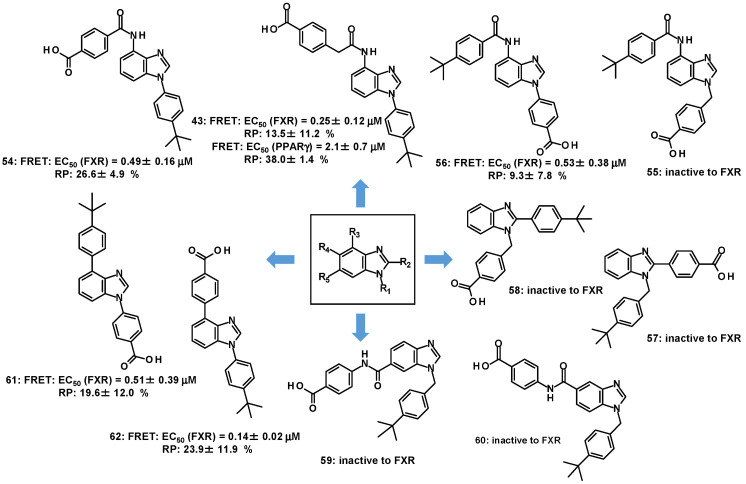
Dual partial agonist (**43**) with benzimidazole for FXR/PPARγ.

As a treatment approach to MASLD, rosiglitazone (**51**), as a full agonist for PPARγ, has shown positive outcomes in a clinical trial involving anti-diabetic agents [83]. (Figure 10) As with the development of FXR agonists, partial agonists for PPARγ such as MRL-24 (**52**) and nTZDpa (**53**) have been investigated [84]. MASLD is closely associated with insulin resistance and T2DM [85]. In this regard, activating both FXR and PPARγ may be able to concurrently address the event relevant to FXR and PPARγ. Since no FXR/PPARγ dual agonists have been reported to date, a benzimidazole-based small cluster was assembled to explore FXR/PPARγ dual agonists. A small compound group consisting of 20 compounds with various substitution patterns on benzimidazole was constructed as well as some benzimidazole analogs (**43**, **54**–**62**), and their efficacies [EC_50_ (μM)] in the FRET binding assay and RP to 2 μM of GW4064 (**1**) are shown in Figure 11 [75]. Partial agonism of FXR in the FRET binding assay was observed for many compounds (**43**, **54**, **56**, **61** and **62**) with substituents at the R1- and R3-positions on benzimidazole but not with other substitution patterns, out of which **43** displayed the EC_50_ value for PPARγ and shared the RP with 2 μM of GW1929 (tyrosine-based PPARγ agonist) in Luc, as indicated in Figure 11 [86].

**Figure 12:**
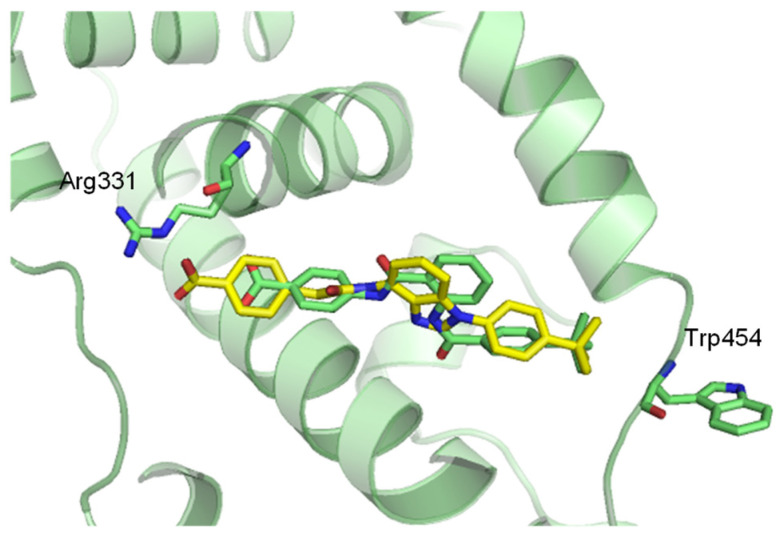
Modeling of FXR complexed with **43**. The complex model was built using FXRɑ complex (PDB ID: 4QE8, green ribbons and sticks) with partial agonist DM175 (**34**) (green sticks) by AutoDock Vina. The dotted line is a hydrogen bond (2.9 Å) between **43** (yellow sticks) and Arg331. Reprinted with permission from Ref. [75].

**Figure 13:**
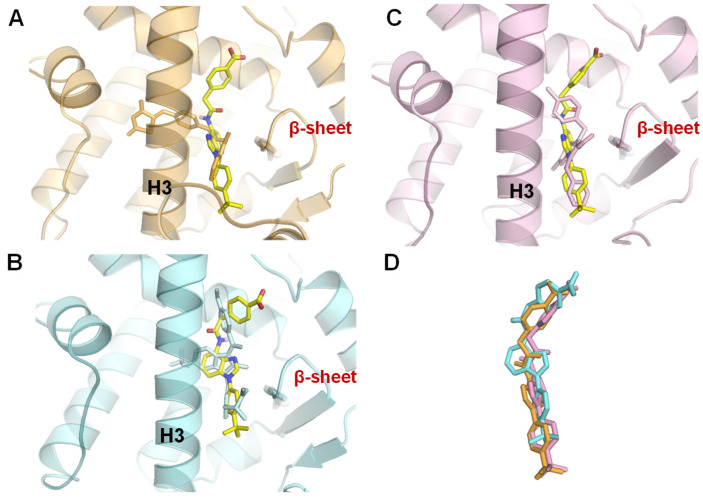
Modeling of PPARγ complexed with **43** (yellow sticks). The complex models were built using three PPARγ complexes by AutoDock Vina (**A**) for complex with full agonist rosiglitazone (**51**) (PDB ID: 2PRG, beige ribbons and sticks), (**B**) for complex with partial agonist MRL-24 (**52**) (PDB ID: 2Q5P, blue ribbons and sticks), (**C**) for complex with partial agonist nTZDpa (**53**) (PDB ID: 2Q5S, magenta ribbons and sticks), and (**D**) superimposing the 3 modeled **43** structures in complex A (orange sticks), complex B (blue sticks), and complex C (magenta sticks). Reprinted with permission from Ref. [75].

A molecular docking simulation (AutoDock Vina 1.1.2) [87] with **43** showed that the acidic moiety in **43** (yellow sticks) can also establish a hydrogen bond with Arg331 like that of DM175 (**34**) (green sticks), as indicated in Figure 6, and the *t*-butylbenzene group of **43** was well overlapped with that of DM175 (**34**). (Figure 12) As discussed in Section 3.2, the *t*-butylphenyl group of **34** flipped Trp454 of FXR to the opposite side. Likewise, **43** also showed a similar trend toward Trp454 as **34**, showing that **43**, like DM175 (**34**), is a partial agonist occupying the LBD, and the disubstituted benzimidazole of **43** functions as a substitute for an anthranilic amide moiety as the core structure of **34**. In contrast, based on the crystal structure of complexes with **51**–**53**, (Figure 10) the binding mode of **43** (yellow stick) is visibly different from that of rosiglitazone (**51**) (beige sticks) (Figure 13A), and the space in which **43** is located between helix 3 and the β-sheet filled by the PPARγ partial agonists **52** (blue sticks) and **53** (magenta sticks) (Figure 13B,C). The same binding modes of **43**, **52**, and **53** are depicted in Figure 13D. It was indicated that **43** has a partially agonistic effect on FXR as well as PPARγ [75].

The anti-diabetic effects of PPARγ partial agonists have been linked with their ability to prevent the cyclin-dependent kinase (CDK) 5-mediated phosphorylation of the PPARγ-Ser273 residue [88]. Pretreatment of mouse 3T3-L1 adipocytes with **43** or rosiglitazone reduced the CDK5-stimulated phosphorylation of PPARγ-Ser273, indicating that **43** inhibited the phosphorylation of PPARγ-Ser273 in a dose-dependent manner. The results suggest that **43** acts as a dual partial agonist for the FXR and PPARγ and has the ability to reduce CDK5-mediated phosphorylation of PPARγ-Ser273, thereby making **43** a feasible candidate for the treatment of MASLD associated with T2DM [75].

The effect of the substituents on the benzimidazole ring varies depending on the position at which they are attached. In particular, the substituent at the 2-position of the benzimidazole is known to have a significant impact on the compound’s profile [89]. When comparing the structures of **33** and **43**, the crucial difference is whether or not they have a cyclopropyl group at the 2-position even though there is a difference of one carbon atom in the substituent at the 4-position, leading to the variation in the biological profiles of both benzimidazole-bearing derivatives (**33** and **43**): selective FXR partial agonist and FXR/PPARγ dual partial agonist, respectively.

Many of the benzimidazole-bearing compounds shown here can generally be synthesized using the methods of Scheme S1, but some require additional effort, as shown below. For the synthesis of **43** and **54**, the method of Scheme S1 cannot be achieved using the method shown in Scheme S1 due to the formation of anticipated undesired product, which makes it impossible to obtain **43** and **54**. Therefore, the use of 2,4,6-trichlorophenyl formate [75] is essential for obtaining the desired compounds (Schemes S2 and S3 in the Supplementary Materials). As shown in Scheme S3, compound **A** was chosen as an intermediate for the synthesis of **43**, but treatment of compound **A** directly in an acid solvent provides the undesired benzimidazole **B**. 2,4,6-Trichlorophenyl formate is a highly reactive crystalline CO surrogate for palladium-catalyzed carbonylation of aryl/alkenyl halides and triflates. The formate was tried as a source of CO, resulting in the key analog (**C**) being obtained by forming a urethane bond between the 2,4,6-trichlorophenyl formate and the primary amine of compound **A**, followed by cyclization in an acid solvent. Thus, the synthesis of **43** and **54** requires the substituted aniline intermediates such as compound **C**, in which case the use of 2,4,6-trichlorophenyl formate as a source of CO yielded benzimidazole derivatives (**43** and **54**) [75].

The challenges associated with designing dual modulators that can target two or more pathways with a single compound are expected to increase. However, these multi-target compounds may open new avenues for future treatment options for metabolic syndromes.

## 5. FXR Antagonists Focusing on Benzimidazole Scaffold

**Figure 14:**
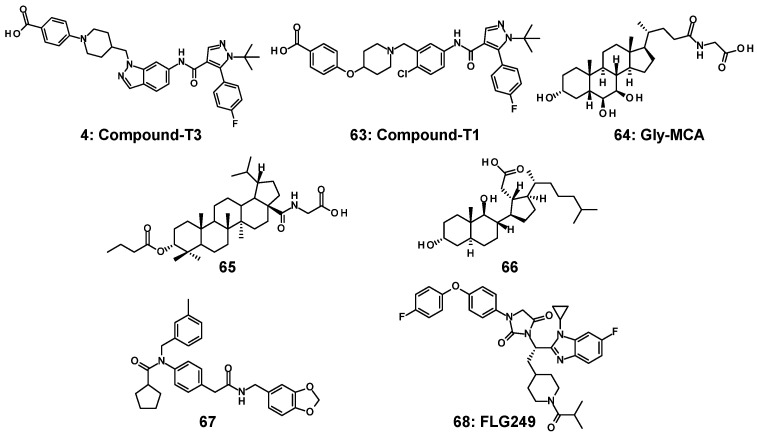
FXR antagonist (**4**, **63**–**68**).

As mentioned in the previous section, the development of agonists is more advanced than that of antagonists in terms of their application in clinical trials of MASH and MASLD. Independent research groups have made significant efforts to develop different FXR antagonists [14,90,91,92,93,94,95,96,97,98]. Of particular interest is compound-T3 (**4**), which was developed by Takeda Pharmaceuticals in animal experiments with cynomolgus monkeys [14]. Compound-T3 (**4**), which possesses indazole as the core structure, activates the cholesterol metabolic system through potent antagonism of FXR, suggesting its potential application in improving dyslipidemia. Subsequently, the anti-dyslipidemic potential of compound-T3 (**4**), along with other non-statin drugs, was investigated in a hamster model of dyslipidemia. In this study, compound-T1 (**63**)—an analog of compound-T3—was also evaluated for comparison [91]. (Figure 14) Unlike compound-T3 (**4**) and compound-T1 (**63**), several reported FXR antagonists lack in-depth biological and pharmacological profiles, including tissue specificity [93,96].

With regard to tissue specificity, intestine-specific FXR antagonists have been subjected to comprehensive pharmacological investigations to elucidate their potential to treat MASH and MASLD. In 2015, Gonzalez et al. disclosed that the conjugated bile acid glycine-β-muricholic acid (Gly-MCA) (**64**), (Figure 14) which triggered the development of intestinal-specific FXR antagonists, selectively suppressed FXR activity in the intestine and promoted browning of beige fat as a result of inhibition of the intestinal FXR–ceramide axis [92]. Ceramide is constantly produced and used as a core structure in the synthesis of complex glycosphingolipids and sphingomyelin. Low levels of ceramide in the intestine may influence steatosis, inflammation, and insulin resistance [99]. Gly-MCA (**64**) reduces blood ceramide levels in the intestine, thereby suppressing lipid synthesis and alleviating fatty liver disease. The results on **64** implied that the inhibition of FXR in the intestine or ileum leads to improved lifestyle-related diseases. Encouraged by the observation of **64**, four research groups have recently and independently reported intestinal or ileum-specific FXR antagonists featuring a variety of core structures: [a] Betulinic acid derivatives with pentacyclic triterpenoids (**65**) were designed as novel chemical entities and intestine-specific FXR antagonists [97]. [b] 9,11-Seco-cholesterol derivatives (**66**), synthesized by cleaving the C ring of cholesterol, inhibited the mRNA expression of the FXR target genes *Shp* and *Fgf15* in the ileum [98]. [c] 4-Aminophenylacetamide derivatives (**67**) prevented intestinal FXR, and oral treatment with **67** showed remedy effects against HFD-induced MASH [91]. [d] Orally active FLG249 (**68**) featuring benzimidazole regulated the mRNA level of three FXR target genes, *Fgf15*, an apical sodium-dependent bile acid transporter (*Asbt*, *Slc10a2*), and *Shp* in mouse ileum [95]. Intestine- or ileum-specific FXR antagonists with various structures have shown promising potential for therapeutic applications in MASH and MASLD.

**Figure 15:**
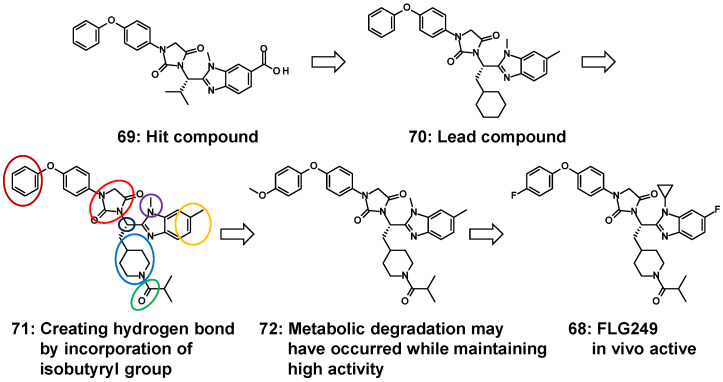
Evolution of analogs with benzimidazole leading up to development of FLG249 (**68**).

**Figure 16:**
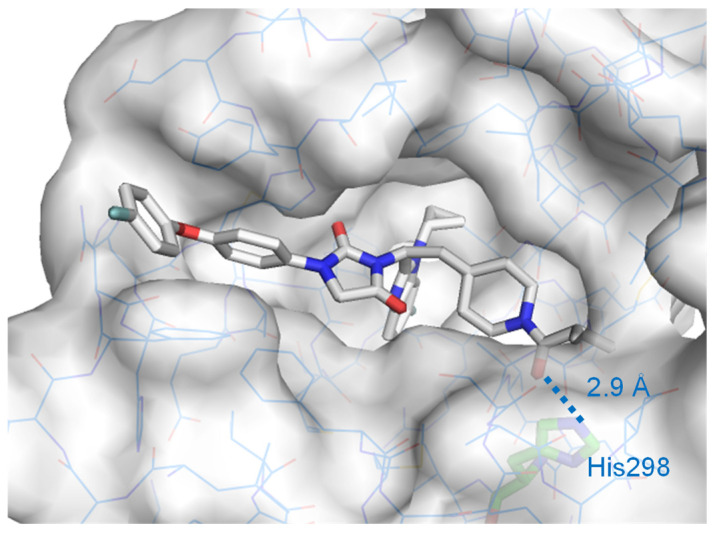
FXR model complexed with **68**. Complex model of hFXRU-LBD homodimer (PDB ID: 4OIV, gray surface) with **68** was built using AutoDock Vina1.1.2. Reprinted with permission from Ref. [108].

In the process leading to **68** containing a benzimidazole core, as depicted in Figure 15, the initial approach began with the identification of hit compound **69** with benzimidazole by means of the in-house compound cluster consisting of approximately one-hundred compounds with benzimidazole [100]. The structural feature of a few active analogs shares amino-acid-derived motifs with an asymmetric carbon between benzimidazole and hydantoin. In the process of derivatization based on the structure of hit compound **69**, two structural pieces on the chemotype were acquired: (i) replacing carboxylic acid with a non-acidic moiety remained active and (ii) introduction of L-cyclohexylalanine alternative to L-valine retained the antagonism. Two alterations were tolerated in LBD of FXR, indicating that a new benzimidazole analog (**70**) offered better antagonistic activity against FXR [100]. Based on the framework of **70**, compound **71**, having N-acyl piperidine, had substantial changes (more than a 1000-fold increase) in its antagonist activity compared to non-acylated compound **70** and reduced triglyceride accumulation in 3T3-L1 adipocytes [101]. Increasing pharmacological or pharmacokinetic parameters at the expense of in vitro activity is often encountered in drug development (e.g., the strategy of medicinal chemistry that led to the histamine H2-receptor antagonist cimetidine [102,103]). It is crucial to consider which regions of **71** can be modified and which cannot. To clarify these regions, the SAR was assessed for seven building blocks (colored circles in Figure 15), including the benzimidazole substituent of **71**. Small substitutions, such as replacing the N-methyl (purple circle) and methyl groups (yellow circle) of benzimidazole, show no substantial changes in the FXR inhibitory activity, and the *p*-substituted benzene ring (brown red circle) in phenoxybenzene is also tolerated by the LBD. Consequently, **72**, with the *p*-substituted benzene, retained the antagonistic activity; however, it showed poor pharmacokinetic profiles, in particular, inferior oral bioavailability and short half-lives and instability in a mouse liver microsome (MLM) assay [104]. In addition, the tissue distribution of **72** was found to be nearly equal between the liver and ileum. The drawback of **72** may be attributed to its instability towards liver microsomes. To address this, metabolically unstable functional groups in **72** [105] were replaced with less susceptible moieties, such as fluorine [106] and cyclopropyl [107]. This led to the development of an orally active compound, namely FLG249 (**68**), which is predominantly distributed in the ileum; however, the antagonistic effect of **68** was attenuated compared to that of **72**. Modeling studies predicted that all moieties consisting of **68** were well tolerated in the LBD, and the carbonyl group of isobutyryl in **68** could establish a network of hydrogen bonds with His298 at 2.9 Å [108]. (Figure 16) The profiles of these key analogs (**67**–**72**) at each step to reach **68** is shown in Table 1. Even short-term administration of FLG249 (**68**) controls the mRNA levels of three FXR target genes, *Fgf15*, *Asbt*, and *Shp*, in the C57BL/6 mouse ileum as FXR target genes. It is noteworthy that replacing three regions in **68** with less metabolically susceptible moieties (such as cyclopropyl and fluorine) improved the stability of MLM and rat liver microsome (RLM) compared to **72**. Similar to the development of cimetidine described above, **68** is also the FXR antagonist obtained by prioritizing metabolic stability while preserving the FXR inhibitory activity as much as possible, and this is due to the exhaustive SAR on this part of **71**. These findings indicate that treatment with **68** resulted in improved pharmacokinetic parameters and had positive effects on the mRNA level of three FXR target genes in the ileum [95].

In addition to the short-term administration study, Iguchi et al. investigated the effects of long-term administration of FLG249 (**68**) in mice fed an HFD for four weeks [109]. FLG249 (**68**)-treated mice did not show any weight loss; however, liver triacylglycerol and blood cholesterol levels significantly decreased. FLG249 (**68**) treatment also altered the expression of genes related to bile acid metabolism, ceramide synthesis, and fatty acid β-oxidation in the liver and ileum. This implies that **68** has the potential to serve as a low-toxicity drug and may improve lipid metabolism disorders as a non-steroidal FXR antagonist [109].

Although the molecular mechanism that partially promotes FXR activation has been inferred by a few groups, as described in Section 3.2 [54,62,63], there have been no reports addressing the antagonistic mechanism of FXR to date. The antagonistic mechanisms of T3 (**4**) and FLG249 (**68**) were compared, and differences in FXR-cofactor interactions were observed [110]. The dose–response curves of **4** and **68** suggested that they are inverse agonists. However, compound T3 (**4**) promoted the recruitment of two corepressors—the silencing mediator of retinoic acid and thyroid hormone receptor (SMRT) and nuclear receptor corepressor 1 (NcoR1) peptides—to FXR. In contrast, **68** inhibited the recruitment of SMRT and NcoR1 peptides to FXR in a dose-dependent manner. The opposing effect of FLG249 (**68**) on corepressors appears to be unusual, and its underlying mechanism remains unexplored [110].

## 6. Perspective: Toward Intestine-Specific FXR Modulators

**Figure 17:**
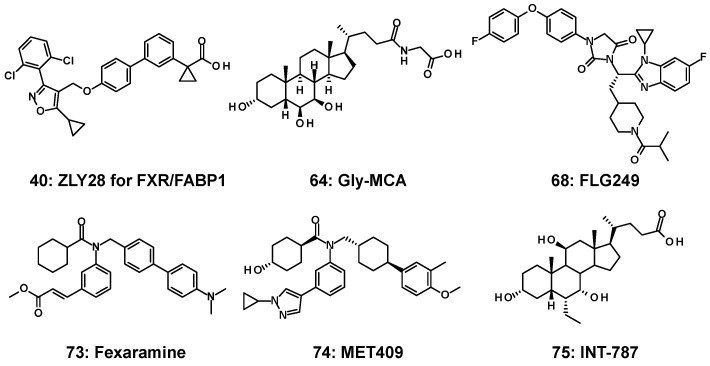
FXR modulators (**40**, **64**, **68**, **73**–**75**) for intestine specificity.

Intestine-specific FXR antagonists have gained attention since Gly-MCA (**64**) was the first compound to be identified as an antagonist of intestinal FXR [92]. (Figure 17) Dorel et al. recently discussed strategies for the development of intestinally restricted drugs [111]. One characteristic of such compounds is that they deviate from the rule of 5 (e.g., molecular weight (MW) greater than 500 g/mol and clogP above 5) [111,112]. For instance, benzimidazole-based **68** (MW: 641.72 g/mol, clogP: 6.49) and **72** (MW: 623.75 g/mol, clogP: 6.30) have similar structural characteristics; however, treatment with **68** (116.45 ± 41.65 μg/g tissue) resulted in an approximately 14-fold higher concentration in the ileum than that with **72** (8.04 ± 1.95 μg/g tissue) [95]. These observations suggest that the optimal range and other factors influencing intestinal specificity are not yet fully understood and warrant further investigation.

In addition, it has been suggested that the intestine-restricted FXR agonist fexaramine (Fex) (**73**) alters the BA pool to contain higher levels of lithocholic acid, causing downstream activation of TGR5, improving energy expenditure and browning of white adipose tissue [113], and it induces effects on enteric FGF19 without activating any target genes in the liver [114]. Given this, intestine-restricted FXR activation has been suggested as a new approach for the treatment of obesity and metabolic syndromes. MET409 (**74**), one of the Fex derivatives, has been investigated in clinical trials. Although the associated patents claim intestinal selectivity, no supporting data have been disclosed [115]. Clinical results showed a reduction in liver fat content and an increase in LDL cholesterol; however, pruritus was reported in 16% of patients [116].

The FXR/FABP1 dual modulator ZLY28 (**40**) also showed intestine-restricted properties and significantly improved the stability of GW4064 (**1**) [72]. Recently, the steroidal FXR agonist INT-787 (**75**), derived from CDCA, was shown to exhibit intestinal localization [117].

Activating or inhibiting intestinal FXR, but not hepatic FXR, may restore lipid homeostasis and ameliorate MASLD; however, this remains controversial. The apparent discrepancy arises from differences in intestinal FXR activity elicited by FXR agonists or antagonists under various pathological conditions. Nevertheless, the use of both FXR agonists and antagonists targeting the intestine has received considerable attention.

## 7. Conclusions

Benzimidazole derivatives are naturally occurring pharmacophores of active biomolecules and are of great importance as scaffolds for chemotherapeutic agents under various clinical conditions. Insights into benzimidazoles provide valuable guidance for medicinal chemists aiming for SAR-informed structural modifications of scaffolds, thereby facilitating drug development for the treatment of various metabolic diseases. To date, medicinal chemists in the FXR research community have adopted various approaches to identify ligands for FXR, such as HTS, hit-to-lead optimization, and structure-based drug design. These approaches have led to the development of numerous FXR modulators whose efficacy is currently being tested in animal models of relevant diseases.

GW4064 (**1**) remains the prime lead compound in drug discovery of FXR modulators. The efforts of the independent research groups with GW4064 (**1**) were successful, which resulted in the development of **2**, **6**, and **7**.

Considering the pleiotropic activity of FXR, partial activation of FXR represents an attractive strategy to avoid the mechanism-based side effects of FXR targeting [118]. The benzimidazole-bearing FXR agonists introduced here (**22**–**26**, **28**–**33**) (Figure 4, Figure 6 and Figure 7) partially activate FXR. Among these, **28** and **29** were tested in LDL-receptor-knockout mice; however, the adverse effects reported for FXR full agonists were not described [53,54]. Whether **22**–**26** and **30**–**33** will yield similar in vivo effects to **28** and **29** is a subject for future investigations. Like full agonists, partial agonists attenuate the FXR–corepressor interaction; however, unlike full agonists, they induce a unique FXR conformation with both coactivators and corepressors, suggesting selective regulation of the target gene [62]. Benzimidazole-containing FXR partial agonists (e.g., **29** and **33**) are likely to remain a focus of active development.

Previous synthetic knowledge of benzimidazole derivatives [119] has provided valuable synthetic strategies for medicinal chemists to make informed structural modifications to the scaffold, contributing to the development of a partial agonist (**29**), an FXR/PPARγ dual agonist (**43**), and the antagonist FLG249 (**68**). Notably, the pharmacokinetic profiles and biological activities of precursors of **29** and **68** were promptly improved by the addition of fluorine to benzimidazole and changing the substituents extending from benzimidazole, resulting in the desired biological and pharmacological activities in animal models.

Although FXR is widely recognized as a key therapeutic target in MASLD, the tissue specificity of FXR modulators and whether FXR modulators activate or inhibit intestinal FXR remain major issues. A wide range of FXR modulators are paving the way for the treatment of lifestyle-related diseases. However, the ultimate success of these modulators must be determined through clinical trial results. There remains strong potential for benzimidazole as a structural motif in FXR modulators, which could advance to future clinical trials.

## Figures and Tables

**Table 1:** Profiles of key analogs in development of **68**.

Cpd.	TR-FRET IC_50_ (μM) Luciferase IC_50_ (nM)	Cytotoxicity IC_50_ (μM) ^2^	Selectivity	Expression Level of FXR Target Genes	Remarks
**69**	174.5 ± 14.5 10,100 ± 800	ND ^1^	ND ^1^	ND ^1^	Hit compound
**70**	126.1 ± 36.9 1.2 ± 0.1	ND ^1^	Selective ^3^	SHP ↑, CYP7A1 ↓	Lead compound
**71**	0.035 ± 0.002 <0.001	>100	Selective ^4^	SHP ↓, BSEP ↓, OSTα ↓	Reducing intracellular TG content in 3T3-L1 adipocytes
**72**	0.007 ± 0.002 <0.001	>100	Selective ^5^	SHP ↓, BSEP ↓, OSTα ↓	Distribution of liver and ileum Bioavailability: 17.99 ± 3.52% Unmodified **72** in MLM (2.4 ± 0.5%) and RLM (12.1 ± 3.9%)
**68**	0.033 ± 0.012 0.05 ± 0.06	>100	Selective ^5^	Ileum: *Fgf15* ↓, *Shp* ↓, *Asbt* ↑ Liver: No effects on *Bsep*, *Shp* and *Cyp7A1*	Ileum-specific Bioavailability: 55.40 ± 2.71% Unmodified **68** in MLM (54.0 ± 4.6%) and RLM (84.4 ± 6.4%) ^1^

^1^ ND: not determined. ^2^ tetrazolium (MTT) colorimetric assay. ^3^ No affinity with RXRα. ^4^ No affinity with RXRα, PPARα, γ, δ, LXRα, β, VDR, and RARα. ^5^ No affinity with RXRα, PPARα, γ, δ, LXRα, β, VDR, RARα, and TGR5. A down arrow indicates a decrease and an up arrow indicates an increase.

## Data Availability

No new data were created.

## Supplementary Materials

The following supporting information can be downloaded at: https://www.mdpi.com/article/10.3390/molecules31030450/s1. Scheme S1: Preparation of **33**; Scheme S2. Preparation of **43** and **54**; Scheme S3. Preparation of dual FXR/PPARg agonist (**43**) using 2,4,6-trichlorophenyl formate.

## Abbreviations

The following abbreviations are used in this manuscript:

FXR	Farnesoid X receptor
NR	Nuclear receptor
BA	Bile acid
CDCA	Chenodeoxycholic acid
CYP7A1	Cholesterol 7α-hydroxylase
SHP	Small heterodimer partner
FGF	Fibroblast growth factor
TGR5	Transmembrane G protein-coupled receptor 5
PBC	Primary biliary cholangitis
MASH	Metabolic-dysfunction-associated steatohepatitis
MASLD	Metabolic-dysfunction-associated steatotic liver disease
LDL	Low-density lipoprotein
UDCA	Ursodeoxycholic acid
T2DM	Type 2 diabetes mellitus
PPAR	Peroxisome proliferator-activated receptor
PXR	Pregnane X receptor
RXR	Retinoid X receptor
AR	Androgen receptor
AF2	Activation function 2
RP	Relative potency
HDL	High-density lipoprotein
TR-FRET	Time-resolved fluorescence resonance energy transfer
Luc	Luciferase reporter gene assay
HTS	High-throughput screening
SAR	Structure–activity relationship
VDR	Vitamin D receptor
MSC	Mesenchymal stem cell
LBD	Ligand-binding domain
SPA	Scintillation proximity assay
GPBAR1	G protein-coupled bile acid receptor 1
LXR	Liver X receptor
LTA4H	Leukotriene A4 hydrolase
FABP1	Intestinal fatty acid binding protein 1
URAT1	Urate transporter 1
sEH	Soluble epoxide hydrolase
HSD17B13	17-β-hydroxysteroid dehydrogenase 13
LIFR	Leukemia inhibitory factor receptor
CDK	Cyclin-dependent kinase
Gly-MCA	Glycine-β-muricholic acid
HFD	High-fat diet
Asbt	Apical sodium-dependent bile acid transporter
RLM	Rat liver microsome
MLM	Mouse liver microsome
SMRT	Silencing mediator of retinoic acid and thyroid hormone receptor
NcoR1	Nuclear receptor corepressor 1
MW	Molecular weight
Fex	Fexaramine
