# Supplementary material for: A Review on Farnesoid X Receptor (FXR) Modulators Focusing on Benzimidazole Scaffold
Source: Molecules. 2026 Jan 27;31(3):450. doi: 10.3390/molecules31030450 (PMC12898554; doi:10.3390/molecules31030450)

Supplementary Materials

A Review on Farnesoid X Receptor (FXR) Modulators Focusing on Benzimidazole Scaffold

Naoki Teno [1,*], Keigo Gohda [2] and Ko Fujimori [3]

1. Faculty of Clinical Nutrition, Hiroshima International University, Kure 737-0112, Japan.

2. Computer-Aided Molecular Modeling Research Center, Kansai (CAMM-Kansai), Nishinomiya 663-8241, Japan.
E-mail: ke.gohda@camm-kansai.org

3. Department of Pathobiochemistry, Faculty of Pharmacy, Osaka Medical and Pharmaceutical University, Takatsuki 569-1094, Japan. E-mail: ko.fujimori@ompu.ac.jp

Content

1. Scheme S1: Preparation of **33**.
2. Scheme S2. Preparation of **43** and **54**.
3. Scheme S3. Preparation of dual FXR/PPARγ agonist (**43**) using 2,4,6-trichlorophenyl formate

1. Scheme S1: Preparation of **33**.

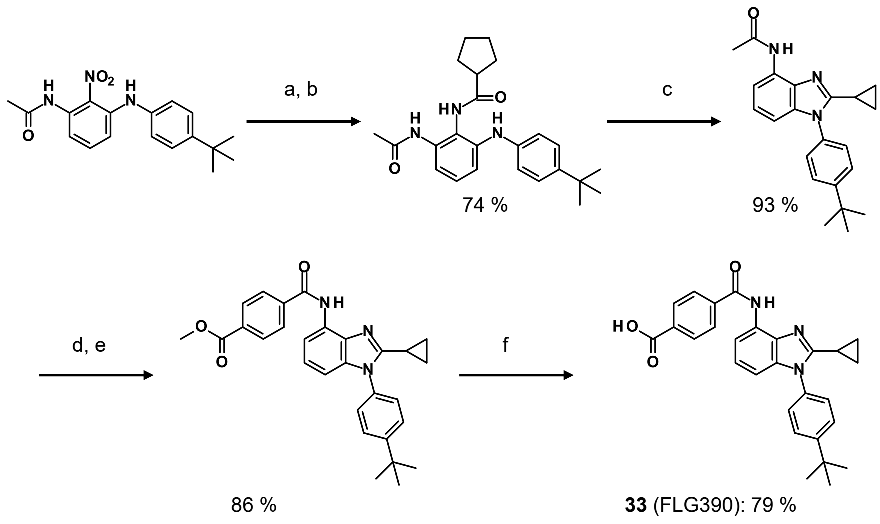

Reagents and conditions: (a) 10 % Pd/C, H_2, MeOH, rt, 1 h; (b) Corresponding carboxylic acid, WSCI.HCl, HOAt, DMF, rt, 15 h; (c) CH_3COOH, 80 °C, 2-4 h; (d) 6 M HCl, Dioxane, 90 °C, 1 h; (e) Monomethyl terephthalate, HOAt, WSCI.HCl, DMF, rt, 15 h; (f) 1M NaOH, THF/MeOH, rt, 15 h.

2. Scheme S2: Preparation of **43** and **54**.

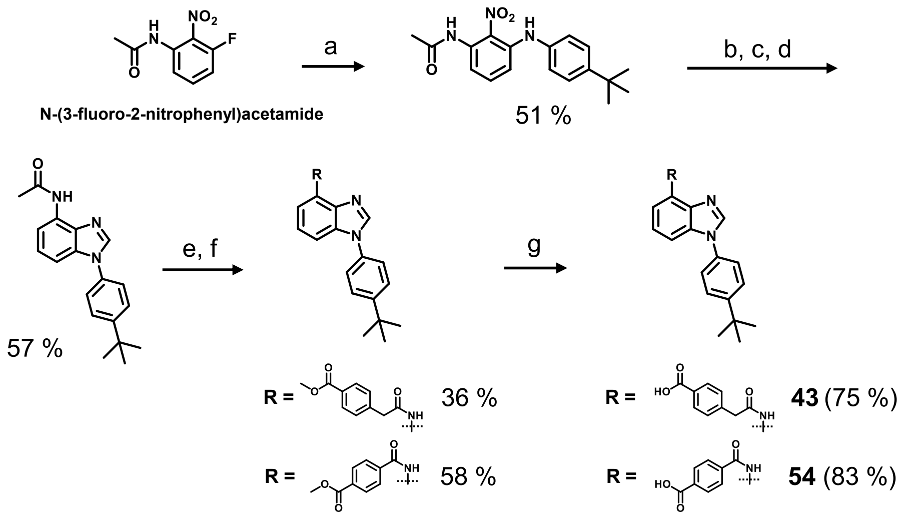

Reagents and conditions: (a) 4-*tert*-Butylaniline, N,N-Diisopropylethylamine, DMSO, 15 h, 120 °C; (b) 10% Pd/C, H_2, MeOH, 2 h, rt; (c) 2,4,6-Trichlorophenyl formate, THF, 15 h, 70 °C; (d) HCOOH, 1 h, 80 °C; (e) 6M HCl, dioxane, 15 h, 90 °C; (f) Corresponding COOH derivatives, HOAt, WSCI· HCl, DMF, 15 h, 0 °C→rt; (g) 1M NaOH, MeOH/THF, 15 h, rt.

3. Scheme S3: Preparation of dual FXR/PPARγ agonist (**43**) using 2,4,6-trichlorophenyl formate

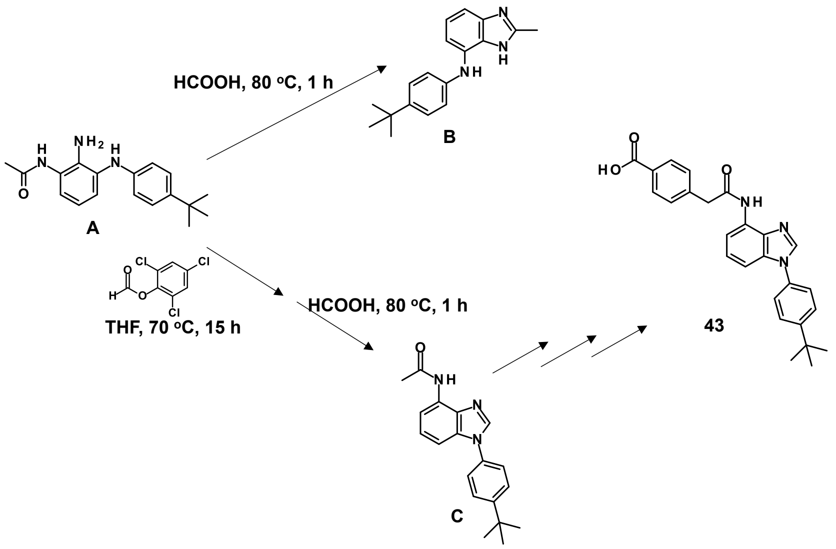

Supplement: Supplementary file 1 [file molecules-31-00450-s001.zip › molecules-4030940-supplementary.pdf]
